# Delineating the Effect of Semantic Congruency on Episodic Memory: The Role of Integration and Relatedness

**DOI:** 10.1371/journal.pone.0115624

**Published:** 2015-02-19

**Authors:** Oded Bein, Neta Livneh, Niv Reggev, Michael Gilead, Yonatan Goshen-Gottstein, Anat Maril

**Affiliations:** 1 Department of Cognitive Science, The Hebrew University of Jerusalem, Jerusalem, Israel; 2 Department of Psychology, The Hebrew University of Jerusalem, Jerusalem, Israel; 3 Department of Psychology, Tel-Aviv University, Tel-Aviv, Israel; Center for BrainHealth, University of Texas at Dallas, UNITED STATES

## Abstract

A fundamental challenge in the study of learning and memory is to understand the role of existing knowledge in the encoding and retrieval of new episodic information. The importance of prior knowledge in memory is demonstrated in the congruency effect—the robust finding wherein participants display better memory for items that are compatible, rather than incompatible, with their pre-existing semantic knowledge. Despite its robustness, the mechanism underlying this effect is not well understood. In four studies, we provide evidence that demonstrates the privileged explanatory power of the elaboration-integration account over alternative hypotheses. Furthermore, we question the implicit assumption that the congruency effect pertains to the truthfulness/sensibility of a subject-predicate proposition, and show that congruency is a function of semantic relatedness between item and context words.

## Introduction

It takes time—but eventually all of us weave, thread by thread, an intricate net of world knowledge. It is clear that we make use of our creation whenever we play a game of trivial pursuit or decide upon the proper ingredients that make a tasty sandwich. However, a less appreciated role of our semantic network is in episodic memory; it appears that we cast this thick net upon the world whenever we capture a new experience. A fundamental challenge in the study of learning and memory is to try and understand the role of existing knowledge structures in the encoding and retrieval of new episodic information.

Much research throughout the years has demonstrated that prior knowledge can facilitate memory. For example, a large body of work shows that experts are better able to remember new information related to their field of expertise than novices (see [1] for a review). Furthermore, the activation of existing knowledge schemas facilitates encoding, as evident in studies that have shown increased retrieval of stimuli that were preceded with short descriptions (e.g., "the paragraph you will now hear will be about washing clothes" [2]). Finally, the importance of prior knowledge in memory is demonstrated in a classic (yet somewhat under-studied) memory phenomenon—the "congruency effect" [3–6]

The "congruency effect" relates to the finding whereby participants display better memory for items that were presented within a context that is compatible, rather than incompatible, with their pre-existing semantic knowledge. For example, participants will display better memory for the target item SPINACH when it is embedded at encoding within the question- "is SPINACH leafy?" (i.e., a congruent statement), than when it is embedded in the question- "is SPINACH ecstatic?" (i.e., an incongruent statement). This effect can be obtained using a broad range of stimuli, and across different modalities (e.g., [3,7–12]). Furthermore, it is observed under both incidental and intentional encoding, and when employing tests of both recall and recognition (e.g., [3,5,13]).

Over the past few years, there has been a resurgence of interest in the congruency effect. This interest has been spurred by research within the field of systems neuroscience (e.g., [4,6,12,14]) and stems from an increasing appreciation of the critical importance of existing knowledge schemas in the successful encoding of new events [15–21],. It seems, therefore, that the congruency effect is reclaiming its place as an imporatnt phenomenon in memory research, probably because it is a straight-forward demonstrations of the general principle of the effects of exisiting representation on episodic memory.

What is the cause of the congruency effect? A prominent interpretation of the memory advantage for congruent items was given in Craik and Tulving's seminal paper on "depth-of-processing" effects [3], which included one of the earliest investigations of the congruency effect. According to Craik and Tulving's *integration-elaboration hypothesis*, the target item (e.g., SPINACH) is assumed to "integrate" more easily with a congruent context (e.g., leafy), as compared to an incongruent context (e.g., ecstatic). Thus, in the congruent condition, the context word is, as it were, "appended" to the target's trace—deeming it more "elaborate". These more elaborate traces are, thereafter, assumed to be more easily accessed during retrieval. This idea is reminiscent of the dual-process models of recognition which posit the existence (alongside a familiarity process) of a recollective—contextually based—process, which supports highly accurate recognition judgments. The idea of a contextual basis of the congruency effect can be contrasted with the notion that the semantically-related words enhance target activation, deeming target items more accessible, regardless of supplementary contextual information (see below, the 'item-strength account').

In the years since Craik and Tulving's paper [3], the important effect of elaboration on memory has been repeatedly demonstrated (for an early review, see [22]). However, the implementation of the integration-elaboration theory to the congruency effect, specifically, the role of context, has remained equivocal. In fact, the central claim, according to which the congruency advantage specifically hinges upon the integration of item and context, has received markedly little empirical investigation.

A *prerequisite* for the viability of this claim is that congruent pairs are indeed better integrated, namely, that a target item should serve as a more efficient retrieval cue for the recall of a previously congruent, as compared to an incongruent, context word. Using a cued-recall paradigm, this prediction was tested and was indeed supported in several studies (e.g., [5,13],[3], Experiment 7; Moscovitch & Craik, 1976; see also [7]). Likewise, reinstatement of the encoding context at test (by the experimenter) facilitated the memory of congruent items more than incongruent items [23], while altering the encoding context during retrieval phase hindered memory of congruent items more than memory of incongruent items ([8], but see [24]), reinforcing the view that components of congruent stimuli are better integrated than those of incongruent stimuli. However, the fact that memory of the context is facilitated for congruent stimuli does not guarantee that herein lies the epicenter of the congruency effect; in other words, the existence of enhanced item-context integration for congruent words provides necessary, but not sufficient, evidence for the integration account.

In fact, recent computational models of human memory [25,26] lend themselves to an alternative interpretation of the congruency effect, whereby the enhanced retrieval of the congruent context is epiphenomenal. According to this alternative account, the congruency of two words displayed at encoding (e.g., "BANANA" and "yellow"), provides an "activation boost" to the target word ("BANANA"). That is, because "BANANA" and "yellow" are bound in our knowledge (whereas "ecstatic" and "SPINACH" are not), activation spreads at encoding from "yellow" to "BANANA", elevating the level of activation of “BANANA” beyond that of simply displaying it at study. This higher level of activation increases the probability that "BANANA" will be remembered better at a later memory test, compared to "SPINACH". According to such a model, then, words appearing in an incongruent context at encoding will have, on average, a lower probability of being remembered at a later memory test. We refer to this account of the congruency effect as the *item-strength* hypothesis.

This item-strength account clearly differs from the item-context *integration* account, whose focus is on the function of the links between target and context items at retrieval (rather than the target’s level of activation at encoding). The integration account suggests that the advantage for congruent words stems from the fact that at retrieval, the congruent context (which is "appended" to the item) is recalled, thus providing additional diagnostic information that increases participants' ability to determine that the item appeared at encoding. As an illustration, if, immediately following the encoding phase, an experimenter could take a pair of scissors and sever the thread that links the words "BANANA" and "yellow"—the retrieval of the congruent target item will still be enhanced according to the item-strength account, but no such enhancement would be expected under the item-context integration hypothesis.

**Fig 1:**
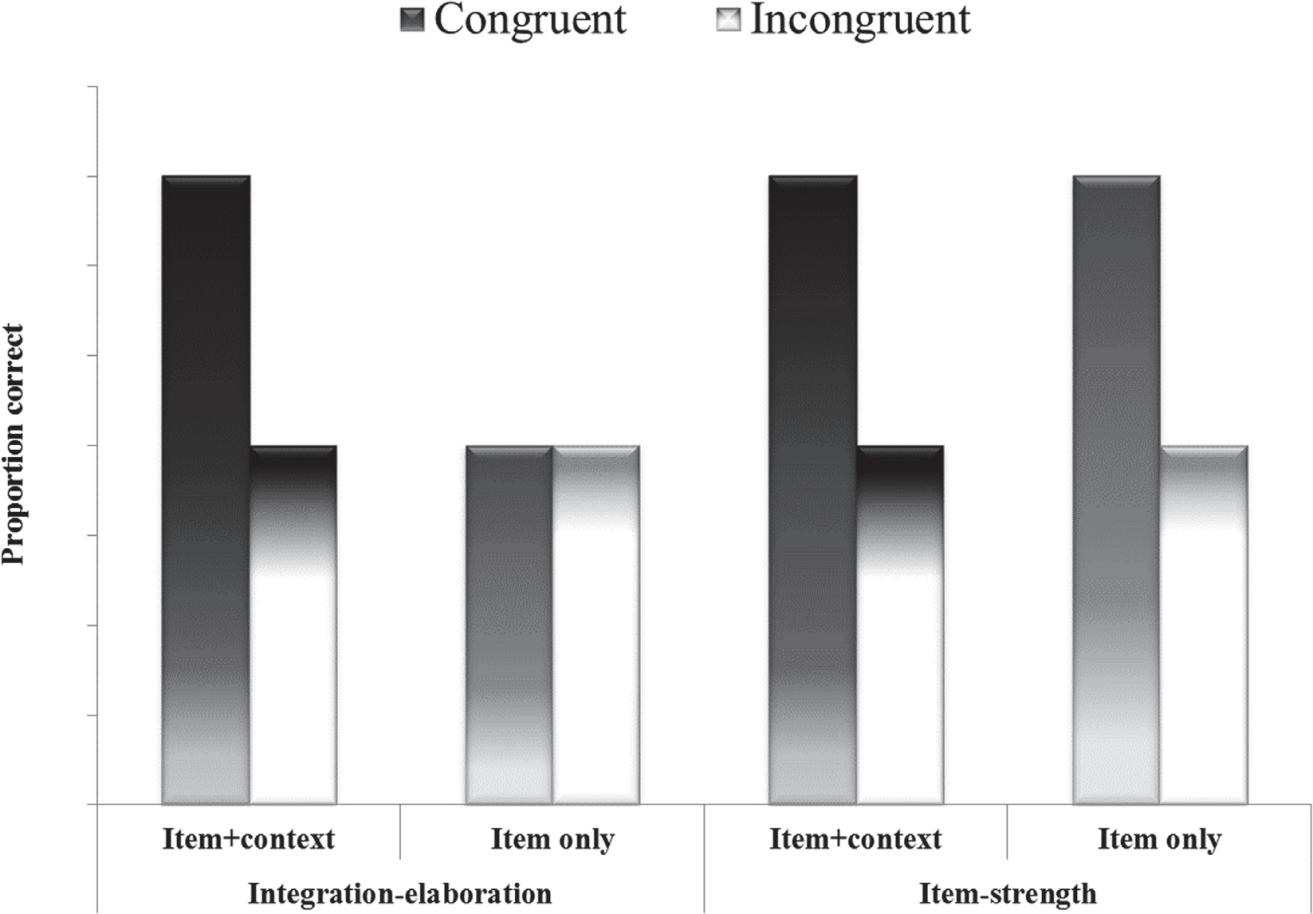
Hypothesized results according to the integration-elaboration account (left side), and the item-strength account (right side). Item+context: target items for which, upon recognition as old, the context word was recalled. Item-only: target items recognized as old without recall of the context word.

To provide a critical examination of the integration-elaboration account of the congruency effect, it still needs to be established that elevated recognition of the target word in the congruent condition can be demonstrated in association with enhanced recollection of context in the congruent, as compared to the incongruent condition. This can be achieved by testing memory for the context word *upon recognition* of the target word. An advantage of preferring this recall-upon-recognition paradigm over cued-recall becomes apparent when considering the following. It is possible that the target word indeed provides an effective cue for retrieval of the context word when the task explicitly requires doing so, (i.e., as in the cued-recall task); yet, when the task is to read the target word and make a decision regarding its study status (i.e., a recognition test—which is a prevalent measure in congruency studies), the target’s effectiveness as retrieval cue may be compromised. That different task demands can lead to divergent mnemonic consequences, even when the identical nominal stimulus is presented to initiate processing, is long known. For example, the implicit-memory literature has revealed that using a cue to consciously recollect information results in different mnemonic consequences than using the identical cue to perform a perceptual task (e.g., [27]). Testing memory for the context word *upon recognition* of the target word would yield four conditions by crossing congruency status (congruent / incongruent) with retrieval status of the context word (yes/no). A pattern of results whereby the congruency advantage is observed in recognition for the target word, regardless of the rate of retrieval of the context word, would be consistent with the item-strength account (see Fig. 1). On the other hand, a pattern of results whereby the congruency advantage for the target word is manifested only in those target items that are remembered with their context word would provide further support for the integration-elaboration view (see Fig. 1).

Thus, the first goal of the current investigation was to test the viability of the integration-elaboration account by examining whether the congruency effect hinges upon enhanced target-context integration. Our subsequent goal was to manipulate the process of integration and to examine whether reducing integration would be accompanied by a reduction or elimination of the congruency effect, as predicted by the integration-elaboration hypothesis. To anticipate our results, our findings garner support to the view according to which the effect indeed stems from enhanced item-context integration.

If item-context integration underlies congruency, then the next question that follows is what underlies item-context integration. Thus, another focus of this study addresses the issue of necessary processes that mediate the integration of an item (e.g., SPINACH) with one context (e.g., leafy) but not with another (e.g., ecstatic). A prevalent implicit hypothesis in the congruency literature (e.g., [3,5,10,28]) is what we refer to as *the proposition hypothesis*. This hypothesis claims that integration in the congruency condition occurs when the truthfulness or the sensibility of a proposition is examined. A proposition is a structured semantic unit which includes a subject (e.g., “SPINACH”) and a predicate (e.g., “leafy”; "SPINACH is leafy"), with the predicate (“leafy”) being a property that characterizes the subject of the proposition ("SPINACH"). Without exception, past research has asked participants to make a decision pertaining to the relationship between a proposition’s subject and predicate, and in doing so, has implicitly adhered to the proposition hypothesis. Importantly, unlike a simple semantic association between two objects (e.g., SPINACH-POPEYE), a proposition asserts a specific logical relation between the subject and a predicate. In doing so, a proposition is a representation of our knowledge of the world that can either be true (e.g., "SPINACH is leafy"), false (e.g., "SPINACH is purple"; "The word spinach rhymes with the word purple"), or insensible (e.g., "SPINACH is ecstatic"). Generally speaking, insensible propositions refer to cases where the predicate belongs to a class of objects that can rarely be attributed to the subject (i.e., mental states to vegetables), whereas false propositions refer to cases where the predicate belongs to a class of objects that are usually attributed to the subject (i.e., color adjectives to vegetables) yet is incompatible with the specific subject.

The proposition hypothesis makes intuitive sense, in that erroneous assertions should not be assimilated into our semantic storehouse. It also coheres with computational models of the 1970's, when the congruency effect was first demonstrated, which were designed to organize our semantic knowledge into propositional relations (e.g., "SPINACH is leafy", or "DOG has legs",[29]). Indeed, these models did not include a representation of false statements (e.g. "SPINACH is not ecstatic").

Still, given that we are able to process and comprehend incongruent propositions, it is not clear why the falsity or insensibility of a proposition should prevent its subject and predicate from forming "a coherent, well-integrated unit" [3]. Moreover, more recent, widely-accepted computational models (e.g., [29,30]), and specifically models which were developed to account for semantic influences on episodic encoding, abandoned propositions as an organizing principle of conceptual knowledge [25,26]. Instead, they proposed that links between nodes in the semantic network are associative, and that they reflect the history of previous co-occurrences of their corresponding concepts. Within such a framework, congruency does not need to rely on a propositional structure. Rather, integration (and hence congruency) is merely a function of the associative connections between nodes in a semantic network. The existence of associative connections between concepts like “spinach” and “leafy’, as compared to “spinach” and “ecstatic”, is therefore predicted to be sufficient to account for the better integration of congruent item-context pairs, without invoking a propositional characterization of these links.

To summarize, the aims of the current work are threefold: (i) to obtain direct experimental support for the enhanced integration hypothesis vs. the item-strength hypothesis (Experiment 1); (ii) to extend the characterization of integration by asking whether this enhanced integration is a factor of truthfulness or semantic relatedness (Experiment 2–3); (iii) to further examine the integration hypothesis by manipulating participants’ ability to perform integration even between two congruent / related concepts (Experiment 4).

## Experiment 1

As noted in the introduction, if the congruency effect indeed hinges upon item-context integration (rather than relying on item-strength), then the benefits of previously congruent targets should manifest themselves specifically in better retrieval of the congruent *context* word.

Prior to examining this critical question, a pilot study was conducted aiming at replicating the classic congruency effect using materials and procedures which will be used in current and later experiments. Congruent/incongruent noun-adjective pairs (e.g., BANANA-yellow, SPINACH-ecstatic; see materials for details) appeared at encoding, while at retrieval only the target noun (e.g., BANANA, SPINACH) appeared for participants to perform an old/new memory judgment. Indeed, this study obtained a congruency effect (Congruent hit-rate: *M* = .66, *SD* = .12, Incongruent hit-rate: *M* = .54, *SD* = .13, *t*(18) = 6.33, *p* < 0.001, one-tailed, Cohen's *d* = 0.95).

To examine memory for contextual information upon recognition of the target word, in the current experiment, we administered the standard congruency-effect protocol, while utilizing a “remember-know” task at retrieval [31–33]. Participants were asked to report whether a positive recognition of the target word was based on retrieval of contextual information from encoding (“remember”) or whether recognition was simply based on a general sense that the item has appeared at study (“know”). If the word did not appear at encoding, they were asked to give a “new” response. We explained to participants that a “remember” response is warranted whenever they felt they remembered some piece of information from the encoding context, be it the context word or any other impression or perception they can recollect. An important modification to the R/K paradigm was that whenever participants made a remember judgment, they were prompted with a screen in which they were asked to report the specific contextual information they remembered. This allowed us to index memory for the context word as well as additional forms of contextual information. Note that our use of the remember-know task was not dependent on a particular theoretical interpretation of this task (e.g., equating "remember" judgments with recollection and "know" judgments with familiarity, see [31–32]); This is because our focus was not on the type of judgment per se (a matter which is still under theoretical debate; see [33–35]) but rather on the ability to actually retrieve the context words or any other contextual information which appeared at study. Furthermore, in our design, failure to retrieve any form of contextual information would not be indexed as memory for context information, even if the trial was categorized as a “remember” trial.

We predicted that target items would elicit more "remember" responses, and, critically, better retrieval of the context word, when appearing at encoding within a congruent proposition than when appearing within an incongruent proposition. Furthermore, if the congruency effect hinges on item-context integration, the congruency advantage should *not* manifest in a higher proportion of "know" responses in the congruent- vs. the incongruent condition (i.e., no difference is predicted between non-integrated items in the two conditions). In line with our prediction, a recent study [36] found enhanced recollection for congruent items compared to incongruent items, in the absence of a difference in familiarity. However, Alberca-Reina et al. [36] presented the entire congruent/incongruent stimuli at test (a pair of faces sharing or not sharing the same profession), and did not test memory for single items, nor did they test the ability of a single item to elicit memory of other components of the stimulus. Therefore, the integration of the stimuli’s components (i.e., integration of the two faces) was not addressed.

### Materials and Method

**Participants**. Across all studies, participants were undergraduate students from the Hebrew University of Jerusalem. They provided a written informed consent to participate in the study, and received either payment of 20–30 NIS (equivalent to 5–8$) or class credit for the participation. All participants were native Hebrew speakers with normal or corrected-to-normal vision. All studies have been approved by the Faculty of Social Science Ethics Committee, The Hebrew University of Jerusalem.

Experiment 1 included 18 participants (8 females; aged 20–28 years; mean age: 23.72). Two additional participants were removed from analysis (one due to poor compliance with the task instructions—2.5 SDs below the mean accuracy rate at encoding; one due to a technical problem).

**Materials**. In all, 360 nouns were matched with congruent and incongruent adjectives to form 720 pairs which were used as target stimuli for this experiment (e.g., the noun “BANANA” could appear with the congruent adjective “yellow” or with the incongruent adjective “purple”). Each adjective that appeared in a congruent pair with one noun (e.g., “yellow BANANA”) was also used to construct an incongruent pair with another noun (e.g., “yellow STRAWBERRY”). Across participants all nouns and all adjectives were seen both in a congruent and in an incongruent pair. The congruent (or incongruent) status of each pair was assessed in a pilot study prior to the experiment. For each participant, 180 pairs (90 congruent, 90 incongruent) were selected for encoding. The remaining 180 nouns were used as lures for the recognition test. Classification of each noun as a target or a lure was counterbalanced across participants as was the pairing of the noun with a congruent or an incongruent adjective. Word frequency was equated across lists using the Hebrew language word frequency database [37].

**General Procedure**. Unless mentioned otherwise, all of experiments were conducted in the following manner. The experiment consisted of three phases: incidental encoding, distraction task, and retrieval. Participants were tested individually. Both the encoding and retrieval phases were preceded with detailed instructions and a short practice session.

In the incidental encoding phase, participants were asked to decide whether the adjective can describe the noun (e.g., "can a BANANA be yellow?"), and to indicate their response by pressing one of two possible keys on a keyboard. This phase included 180 noun-adjective pairs (90 congruent and 90 incongruent pairs). Each pair was presented on screen for 1.5-s, followed by a 0.5-s fixation cross. Participants could respond during the presentation of the stimuli and the fixation. The pairs appeared simultaneously in the center of the screen, written in white on a black background. The nouns in these pairs always appeared above the adjective (as the standard reading order in Hebrew is a noun followed by an adjective). The pairs appeared in a random order, with a restriction that no more than 4 pairs of a given condition could appear sequentially.

Following the encoding phase, participants were engaged in a visual block-counting task for 5 minutes, to prevent a ceiling effect in memory performance.

The last phase consisted of a surprise memory test. The target words appeared alone in the middle of the screen (180 from the encoding phase and 180 foils). The words were presented on the screen for 3-s followed by a 1-s of fixation. Participants had to choose between three response options by pressing one of three corresponding buttons. The response options were: the word did not appear in the previous part (a "new" response; N); the participants know that the word appeared but could not retrieve any additional information about the encoding event (a "know" response; K); or they know that the word has appeared and they remember additional information about the encoding event (a "remember" response; R). If a participant pressed "remember" s/he was asked to type the information that came to mind. Participants were instructed that additional information can be the context word or anything they remember happening, or thinking of, while seeing the word in the first part of the experiment. The participants were also given a few examples of what such information might be. The items were presented in a random order, with the restriction of no more than 4 items of the same class (old/new) could appear consecutively. At the end of the experiment participants were debriefed and asked whether they had suspected a later memory test.

### Results

Accuracy rates at the incidental encoding task did not differ between congruent and incongruent items (related: *M* = .92; unrelated: *M* = .91, *t*(17) = .23, *n*.*s*.). We excluded from the analysis “remember” responses in which the word typed by the subject was erroneous (.01 of the responses).

R rates were calculated from total number of responses per congruency condition. To correct for possible dependency between R and K responses, corrected K were computed as the percentage of K responses from the number of total responses subtracted by number of R responses (corrected K = K/(total number of responses-R), [32]).

We first examined the effect of congruency on "total memory" (“remember” and “know” responses collapsed together). The results show that overall memory performance was better for congruent items (congruent: *M* = .67, *SD* = .11; incongruent: *M* = .60, *SD* = .16; *t*(17) = 3.27, *p* = .001, one-tailed, Cohen's *d* = 0.58). Mean false alarms rate was .25 (*SD* = .16).

**Fig 2:**
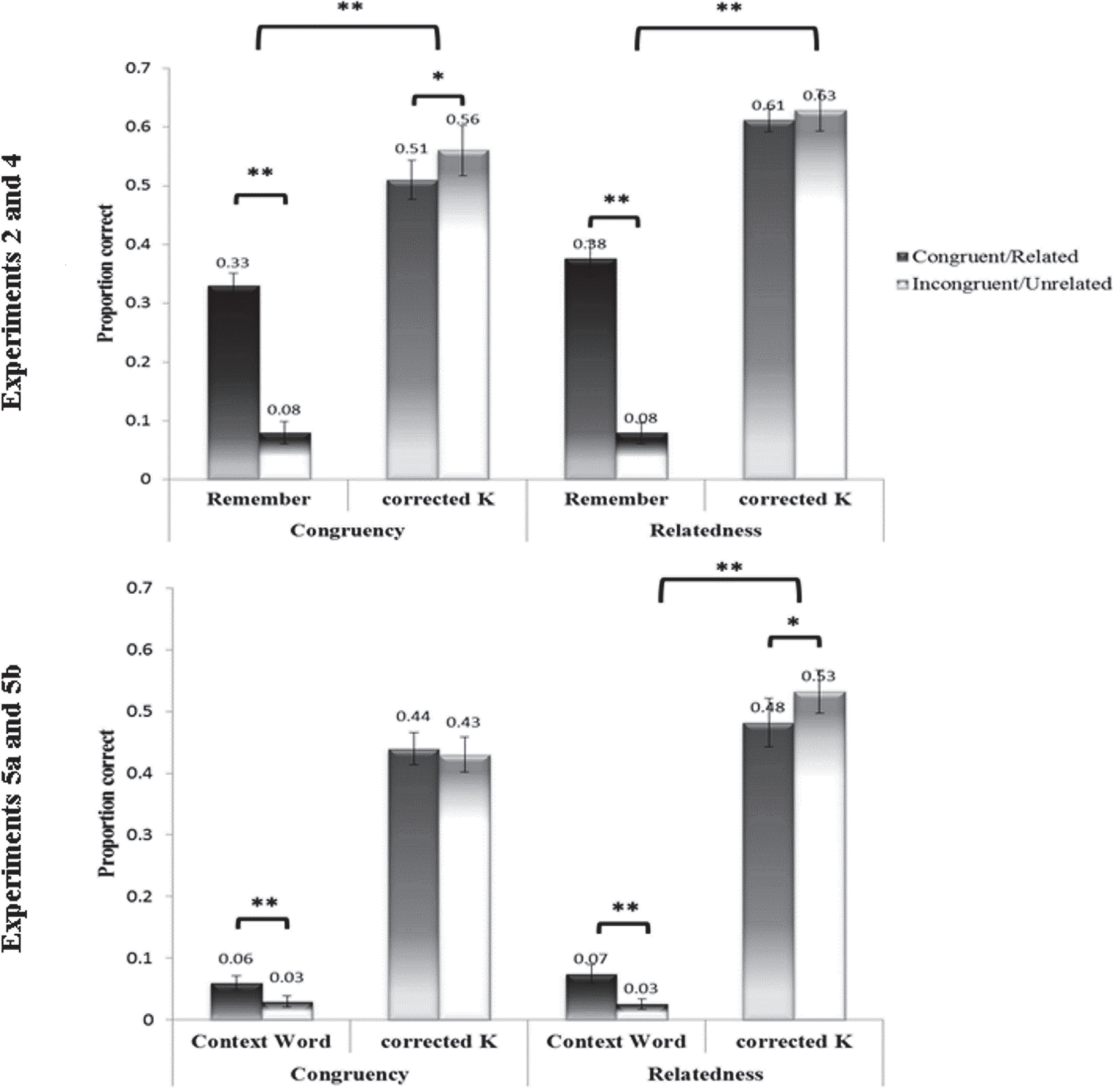
Remember and corrected K rates in Congruency and Relatedness. Upper panel: Experiments 1 and 3, in which participants examined the semantic relations between the components of the stimuli. Lower panel: Experiments 4a and 4b, where participants examined the lexical status of the items (nouns or adjectives).

As predicted by the integration-elaboration hypothesis, R responses-rates were higher for congruent items than for incongruent items (congruent: *M* = .33, *SD* = .09; incongruent: *M* = .08, *SD* = .08). Also as predicted, we did not find a higher K response rate for the congruent condition. In fact, the effect was in the opposite direction (congruent: *M* = .51, *SD* = .14; incongruent: *M* = .56, *SD* = .18; see Table 1 and Fig. 2). A 2 (Congruency: Congruent, Incongruent) by 2 (Judgment: Remember, Corrected K) repeated measures ANOVA revealed a significant interaction of Congruency and Judgment, *F*(1,17) = 75.5, *MSE* = .005, *p* < .001. Simple effects tests confirmed that the R differences were reliable, *t*(17) = 13.47, *p* < .001, one-tailed, Cohen's *d* = 3.26, as well as the corrected K differences, *t*(17) = 2.14, *p* < .05. The majority of R responses were ones in which the context word was recollected unaccompanied by additional information (84% of R responses), while reporting of details in addition to the context word was rare (16%, which translates to roughly 4 items per subject).

**Table 1:** Proportions of Remember and corrected K responses for congruent and incongruent items in Experiment 1 (standard deviations are given in parentheses).

	Remember	Corrected K
Congruent	.33 (.09)	.51 (.14)
Incongruent	.08 (.06)	.56 (.18)

Paired-sample t-test revealed that RT's at encoding were longer for incongruent items than for congruent items (congruent: *M* = 1225 ms, *SD* = 157 ms, incongruent: *M* = 1357 ms, *SD* = 212 ms; *t*(17) = 3.16, *p* < .01; similar results were obtained in the pilot study described in the introduction to the current experiment). Hence, enhanced memory for congruent items cannot be attributed to longer processing time at encoding.

### Discussion

The classic congruency effect is characterized by better memory for congruent items than for incongruent items. In the current experiment, we teased apart the mnemonic advantage of congruent items into two constituent elements: memory for target item only ("know" response) and the ability to recall the context word ("remember" response). As predicted by the integration-elaboration account, participants were better able to recall a congruent (vs. incongruent) context word, indicating that congruent pairs were indeed better integrated.

Our result is consistent with previous findings that have shown that congruent items facilitate retrieval in cued-recall paradigm ([3], Experiment 7; [5,13]). We extend this past work by showing that the enhanced item-context integration is evident also in the case of a recognition paradigm. Furthermore, and perhaps most critically, the results show that item-context congruency was specific to integrated items, and did not result in increased item-only memory, as predicted by the item-strength account (see Introduction and Fig. 1). Therefore, the results suggest that the overall higher level of congruent item recognition observed in the classical congruency paradigm stems from an enhanced ability to recollect the context word. This implies that the facilitated item-context integration is not merely epiphenomenal, but rather constitutive of the congruency effect.

## Experiment 2

Experiment 1 provided support to the view according to which the congruency effect hinges upon the integration of target item and context. As a second step in our investigation of the congruency phenomenon, we wished to delineate what it is about congruent statements that facilitates item-context integration.

Previous demonstrations of the congruency effect were conducted under an implicit assumption we referred to as the "proposition hypothesis". Participants were presented with true/sensible and false/insensible subject-predicate propositions. For example, as was shown in Experiment 1, participants had to decide whether an adjective ("yellow"/"purple") can describe a noun ("BANANA"). In these studies, congruent and incongruent statements differed from each other along two dimensions: (i) congruent statements formed true and sensible subject-predicate propositions while incongruent statements did not (e.g., SPINACH is indeed leafy, but is rarely ecstatic); (ii) items that were embedded within a congruent statement were semantically related to their context, whereas in the incongruent case they were not (e.g., SPINACH and leafy are associated within one's semantic network, whereas SPINACH and ecstatic are not). Based upon these previous studies it remained unclear whether it is the falsity of a statement that disrupts target-context integration, or whether this integration is facilitated by the semantic relatedness of the target and context words.

Therefore, in Experiment 2, we aimed to investigate whether the congruency effect pertains only to true/sensible (vs. false/insensible) subject-predicate propositions, or whether it would also emerge for related (vs. unrelated) word-pairs (e.g., "BIKINI-beach" vs. "BIKINI-leaves"). Unlike the proposition "ecstatic SPINACH" which violates our semantic knowledge of spinach (and is therefore insensible/false) the simultaneous presentation of the words "BIKINI" and "leaves" or "beach" does not form a proposition that is either true or false; instead, it merely differs with regards to the degree of semantic relatedness between the target and context words. To allow a straight-forward comparison with previous demonstrations of the congruency effect, we will first examine the relatedness effect using an old/new recognition test (Experiment 3 will use a “remember-know” memory test). A finding whereby the mere semantic relatedness between item-context pairs leads to enhanced recognition will imply that incorporating the "proposition hypothesis" into an account of the congruency effect is un-parsimonious.

Note that such a comparison of memory of related and unrelated pairs is different from that examined in typical studies of recognition memory for associations. In those studies (e.g., [38]), participants are presented with related and unrelated pairs at study. At test, intact pairs (“old”)—both related and unrelated—or re-combinations of pairs (“new”)—both related and unrelated—are presented for a recognition test. Thus, two items are presented at test, with the items either having been studied within the same pair or in different pairs. Consistent with our suggestion, the robust finding in this literature is that recognition memory for related pairs is better than for unrelated pairs. Presumably, the appearance of each of the studied words at test alongside the other pair member enhances the recollection for the other word, perhaps by some process of co-activation. In contrast, in the current experiment, we present only a single item at test (the target item). Historically, this item may have appeared as part of a related pair or as part of an unrelated pair. Critically, this information is long-gone and is not present when making the memory judgment. If the study status of the item (related, unrelated) leads to differential recognition memory, it will provide a demonstration of the congruency effect in a setting that does not support the proposition hypothesis.

### Materials and Method

**Participants**. Twenty four participants (9 female; aged 20–29 years old; mean age: 25) participated in the study. Two additional participants were excluded from analysis since they suspected a later memory test.

**Materials**. In all, 456 pairs of related words and 456 pairs of unrelated words were used in this experiment. Several types of semantic relations were included in the stimuli list: abstract and concrete nouns (e.g., LOVE—affection; CHAIR—table), oppositions (e.g., LIGHT—dark), members of the same category (e.g. DOG-cat) and items that are not members of the same semantic category but tend to appear together in our daily life (e.g., MOTORCYCLE—helmet; BIKINI-beach; WALL-picture). To compose the unrelated pairs, we interchanged the context words between two target words. For example, the related pairs CHICKEN-egg and NECKLACE-earrings were interchanged to make the unrelated pairs CHICKEN-earrings and NECKLACE-egg. Related and unrelated pairs were chosen based on associative connection as assessed in a pilot study, in which participants rated the relatedness of word pairs.

Pairs were divided into four lists, composed of related and unrelated pairs (228 pairs in each list, 114 related and 114 unrelated) that rotated across participants. The pairing of a noun with a related noun or an unrelated noun was counterbalanced across lists. All lists were equated for relatedness score. Word frequency was equated across lists using the Hebrew language word frequency database [37]. Across participants, all the nouns were seen both in a related and in an unrelated pair. The classification of each word as a target or a lure was counterbalanced across participants.

**Procedure**. At encoding, the 228 pairs (114 related pairs, 114 unrelated pairs) appeared on screen for 2.5-s, followed by 0.5-s of fixation. The words appeared simultaneously at the center of the screen; the target word was always above the context word. Participants were asked to indicate whether the two presented words are related or unrelated in their meaning by pressing one of two keys on a keyboard.

On the recognition test, which immediately followed the encoding phase, all the target words were presented again alone on the screen (228, half previously related and half previously unrelated) intermixed with new words (228 items). Each word was presented for 1.5-s, followed by a 0.5-s fixation.

### Results

At encoding, .95 of the related trials and .94 of the unrelated trials were classified by the participants in accordance with our a-priori classification, *t*(23) = .55, *n*.*s*.

As predicted, average hit rate for items that appeared at study with a related context word (*M* = .61, *SD* = .14) was higher than for items appearing with an unrelated context word (*M* = .47, *SD* = .14), *t*(23) = 10.11, *p* < 0.001, one-tailed, Cohen's *d* = 1.00. Mean false alarms rate was 0.16 (*SD* = .09).

Consistent with our findings in Experiment 1, here too RT’s at encoding were significantly longer for unrelated items than for related items (related: *M* = 1124 ms, *SD* = 197 ms; unrelated: *M* = 1303 ms, *SD* = 249 ms; *t*(23) = 6.33, *p* < .0001).

### Discussion

The results showed that a word will be better remembered after appearing at encoding with a semantically related (vs. unrelated) word (For additional investigations of the effect of semantic relatedness see, for example, [39–41]. The results of these studies are consistent with our findings; however, they do not pertain to the congruency effect). Like in the pilot study and in Experiment 1, this memory advantage cannot be attributed to longer processing time at encoding. Furthermore, similarly to our pilot study using congruency stimuli, the results showed a large effect size of about one standard deviation between the related and unrelated conditions. Importantly, these results were obtained using a paradigm similar to the classic paradigm used to demonstrate the congruency effect, in which the recognition test presents a single item (with no reference to its semantic status at encoding). Therefore, unlike previous studies which tested memory for the associated pair (e.g., [38]), our results cannot be easily attributed to a semantic relatedness effect during retrieval. Thus, the results of Experiment 2 provide support to the claim that the congruency effect stems from the mere semantic relatedness between congruent items and that the "proposition hypothesis" may be unnecessary in order to explain the enhanced item-context integration of congruent pairs.

## Experiment 3

Experiment 2 provided evidence that the mere semantic relatedness between an item and a context might be sufficient to account for the congruency effect. In Experiment 3, we wished to further substantiate this claim by investigating whether the enhanced mnemonic performance for semantically related words mirrors the pattern of results observed in Experiment 1 for propositional stimuli. That is, we wished to see whether the relatedness leads to enhanced recollection of the context word, without an increased retrieval of the target item unaccompanied by its context. Therefore, Experiment 3 provided a replication and extension of Experiment 1, wherein we made use of semantically related rather than subject-predicate propositional stimuli.

### Materials and Method

**Participants**. Sixteen participants (10 females; aged 20–33; mean age: 24) participated in this experiment. Three additional participants were removed from analysis since they suspected a later memory test.

**Materials**. The same stimuli as in experiment 2 were used in this experiment.

**Procedure**. The encoding phase was identical to that of experiment 2, with the exception that the stimuli appeared on screen for 3-s and followed by 0.5-s of fixation.

On the recognition test, which immediately followed the encoding phase, the target words appeared alone on in the middle of the screen (228 from the encoding and 228 foils). The words were presented on the screen for 3-s followed by 1-s of fixation. The task performed by the participants was identical to the one used in Experiment 1 (i.e., a modified remember-know paradigm). The items were presented in a random order, with the restriction of no more than 4 items of the same class (old/new) appearing consecutively.

### Results

At encoding, accuracy rates were equal for related and unrelated items (related: *M* = .95; unrelated: *M* = .97, *t*(15) = .61, *n*.*s*.). Items that were not classified in accordance with our a-priori classification, or items for which the participant did not respond, were excluded from analysis. We also excluded from the analysis “Remember” responses in which the word typed by the participant was erroneous (only .01 of the responses were of this type).

We first wished to see whether the effect of higher memory for related versus unrelated pairs was replicated in this study. Total memory (R and K collapsed together) for related items (*M* = .74, *SD* = .9) was indeed facilitated compared to unrelated items (*M* = .62, SD = .12), *t*(15) = 4.48, *p* < .001, one-tailed, Cohen's *d* = 1.13. Mean false alarms rate was .24 (*SD* = .13).

Consistent with our prediction, R rates were higher for related items than for unrelated items (related: *M* = .37, SD = 12, unrelated: *M* = .08, *SD* = .07) while corrected K rates did not differ as a function of relatedness (related: *M* = .61, *SD* = .08, unrelated: *M* = .63, *SD* = .14; see also Table 2 and Fig. 2). A repeated measures ANOVA with Relatedness (Related, Unrelated) and Judgment (R, corrected K) as independent variables, confirmed the interaction of Relatedness and Judgment to be significant, *F*(1,15) = 58.33, *MSE* = .007, *p* < .001. A simple effects analysis affirmed the difference in R to be reliable, *t*(15) = 9.83, *p* < .001, one-tailed, Cohen's *d* = 3.32, while corrected K rates did not differ, *t*(15) = .56, *p* = .59.

**Table 2:** Proportions of Remember and corrected K responses for related and unrelated items in Experiment 3 (standard deviations are given in parentheses).

	Remember	Corrected K
Related	.37 (.12)	.61 (.07)
Unrelated	.08 (.07)	.63 (.14)

As in Experiment 1, the vast majority (86%) of R responses were ones in which participants were able to recollect the context word, but without further contextual details. Also in this experiment, RT's at encoding were longer for unrelated than for related pairs (related: *M* = 1210 ms, *SD* = 190 ms; unrelated: *M* = 1420 ms, *SD* = 265 ms; *t*(*15*) = 3.99, *p* < .005), indicating that memory differences cannot be explained by longer processing time at encoding.

### Discussion

In Experiment 3, participants were presented at encoding with semantically related (or unrelated) word pairs. At retrieval, participants were asked to judge whether the target item was familiar or not, as well as to attempt to retrieve the context word. Unlike in Experiment 1, the stimuli in this study were non-propositional, and yet, just like in Experiment 1, the effect of semantic relatedness was manifested specifically in enhanced recollection of the context word without a corresponding increase in the target item's familiarity. Thus, the results of this experiment further support the claim that the "proposition hypothesis" is unnecessary to account for the congruency phenomenon. Furthermore, the results provide further support for the claim that the congruency effect specifically hinges upon enhanced item-context integration.

## Experiment 4

In Experiments 1 and 3, we found that the congruency effect is characterized by an enhanced recollection of the context word, without an enhanced recognition for non-integrated target items. If the congruency effect indeed hinges upon the enhanced item-context integration, then a manipulation that specifically disturbs item-context integration should also diminish the magnitude of the congruency effect or altogether eliminate it. If, as suggested by the item-strength account, item-context integration is epiphenomenal to the congruency effect, a congruency effect is likely to be observed in the face of decreased integration.

Previous studies have shown that in order for participants to be able to associate and integrate two items in explicit memory, they must process the relationship between the two words (see, for example, [42,43]). Therefore, unlike in Experiments 1–3 wherein participants’ encoding task involved the processing of semantic relations (i.e., either verifying propositions or assessing relatedness), in Experiment 4 we asked participants to merely determine whether stimuli comprised of two nouns or a noun and an adjective, a task that necessitated the processing of each word but not of the relation between the two. At retrieval, we again employed a modified remember-know task (see Methods). We predicted that the memory for context word will diminish under these non-relational conditions, but that the target item's familiarity would remain relatively unaffected. Furthermore, if the congruency advantage relies upon item-context memory, we predict that total memory difference between congruent and incongruent items would decrease as well.

In Experiment 4A, we used the subject-predicate stimuli employed in Experiment 1; in Experiment 4B, we used word pairs that did not comprise a subject and a predicate, namely, the stimuli employed in Experiment 2 and 3.

### Experiment 4A

**Materials and Method. Participants**. Eighteen participants (13 females, aged 23–31; mean age: 25.17) participated in this experiment. One additional participant was excluded from the analyses due to poor performance at the encoding task (more than 2.5 SDs below the mean).

**Materials**. The noun-adjective stimuli, which were in the focus of this experiment, were a subset of 480 pairs from the ones used in Experiment 1 (these were divided to four lists as was done in Experiment 1: 60 congruent and 60 incongruent pairs appeared for each participant at encoding, and additional 120 words appeared as foils at test). To allow the two-nouns/noun-adjective task (see procedure), two additional sets of stimuli were added to this experiment as fillers: 24 noun-adjective pairs, 12 congruent and 12 incongruent, were constructed for presentation whereby the adjective on the top and the noun at the bottom, and additional 42 pairs composed of two nouns. These sets of pairs were not counterbalanced and were not used in the analyses.

**Procedure**. At encoding, participants were asked to indicate whether a pair of two nouns or a pair of noun and adjective appeared on screen. In the experimental set of stimuli, the target noun always appeared above the context adjective. Additional set of noun-adjective pairs in which the adjective appeared above the noun where used, thus the participants could not predict where the adjective will be and fixate on one word to make a decision. The pairs appeared on the screen for 1.5-s, followed by a 0.5-s fixation.

The retrieval task was similar to that of Experiments 1 and 3. In those experiments, we found that when participants gave a “remember” response they invariably remembered the context word. Moreover, participants were highly accurate in their retrieval of the context word. Therefore, in this experiment two changes were introduced: first, the “remember” response option was replaced by a “context word” (CW) response. Participants were asked to press CW if they remembered that the target word appeared in the previous part, and if they could also retrieve the context word of the pair. Second, in this experiment, we did not ask participant to type in the context word. Instead, participants were merely asked to press CW and were asked to make sure they were capable of saying the word to themselves. If participants remembered that the presented word had appeared, but could not recollect the additional word of the pair, they were asked to press “know” (K). If participants thought that the word did not appear previously, they were to press on “new” (N). All the target nouns from the encoding phase were presented alone in the middle of the screen intermixed with new nouns. Each noun was presented for 2.25-s, followed by a 0.75-s fixation. The order of the items was random, with the restriction of no more than 4 items of the same class (old/new) could appear consecutively.

**Results**. Mean accuracy rates of critical trials (noun-adjective pairs) at the encoding task were higher for congruent pairs (*M* = .85) than for incongruent pairs (*M* = .81), *t*(17) = 2.38, *p* < .05. Pairs whose classification was not in accordance with our experimental pre-classification were excluded from further analyses.

“Total memory” (CW responses with K responses) did not differ between congruent (*M* = .47, *SD* = .12) and incongruent items *(M* = .45, *SD* = .12), *t*(17) = .73, *p* = .48. Mean false alarm rate was .24 (*SD* = .13). CW and corrected K rates were entered to an ANOVA with Judgment (CW, corrected K) and Congruency (congruent, incongruent) as independent variables. This ANOVA revealed no interaction of Judgment and Congruency (*p* > .27). Nevertheless, to examine our hypothesis regarding CW and corrected K rates, simple effect tests were conducted for each judgment (as was done in Experiments 1 and 3). Congruent items (*M* = .06, *SD* = .05) elicited significantly more CW responses than Incongruent items (*M* = .03, *SD* = .04), *t*(17) = 3.55, *p* = .002, Cohen's *d* = 0.66. The proportion of K responses did not differ for congruent (*M* = .44, *SD* = .11) and incongruent items (*M* = .43, *SD* = .12), *t*(17) = .22, p = .82, see Fig. 2.

Incongruent items took significantly longer to process than congruent items (congruent: *M* = 1168 ms, *SD* = 184 ms, incongruent: *M* = 1223 ms, *SD* = 184 ms; paired-sample t-test: *t*(17) = 5.27, *p* < .001).

Thus, when we produced a disturbance of integration, a reduction in memory performance was specifically manifested in the context-word retrieval, thereby eliminating the congruency advantage.

### Experiment 4B

**Materials and Method. Participants**. Eighteen participants (9 female; aged 22–31; mean age: 24.4) participated in this experiment. One subject was removed from further analysis due to poor performance (more than 2.5 standard deviations below the average).

**Materials**. A subset of 280 related and 280 unrelated pairs was chosen from the stimuli used in experiment 2. These were divided into 4 lists as was done in experiment 2. For each participant, one list of pairs was selected to be presented at study (70 related pairs and 70 unrelated pairs) while the remaining target words (140 words) were used as lures at the recognition test. Allocation of each word to target or lure, and the pairing of a word with a related or an unrelated context word were counterbalanced across participants.

Another set of 88 pairs of nouns and adjectives, half related and half unrelated was incorporated in the experiment. These pairs were included to enable the task (see procedure, Experiment 4a). They were not counterbalanced; they did not appear at the retrieval phase and were not analyzed.

**Procedure**. The procedure was identical to that of Experiment 4a, only that retrieval test immediately followed encoding.

**Results**. Accuracy rates of encoding task were higher for related items (*M* = .94, *SD* = .04) than for unrelated items (*M* = .90, *SD* = .09), *t*(17) = 3.17, *p* < .01. Pairs whose classification was not in accordance with our experimental pre-classification were excluded from further analyses.

Consistently with our hypothesis, no difference was found between related and unrelated items in the "total memory" measure (related: *M* = .53, *SD* = .17; unrelated: *M* = .54, *SD* = .14 *t*(17) = .53, *p* = .61). Mean false alarm rate was .28 (*SD* = .12). To further examine our hypothesis, CW and corrected K rates were entered to a repeated-measures ANOVA with Judgment (CW, corrected K) and Relatedness (relate, unrelated) as independent variables. This ANOVA revealed an interaction of Judgment and Relatedness (*F*(1,17) = 18.05, *MSE* = .002, *p* < .001). Simple effects tests showed that this interaction stemmed from better recall of CW for related items than for unrelated items (Related: *M* = .07, *SD* = .06; unrelated items: *M* = .03, *SD* = .04, *t*(17) = 4.21, *p* < .001, Cohen's *d* = 0.78). Corrected K responses were higher for unrelated items (*M* = .53, *SD* = .15) than for related items (*M* = .48, *SD* = .17, *t*(17) = 2.65, *p* = .02, see Fig. 2).

RT's at encoding were longer for unrelated items than for related items (related: *M* = 1314 ms, *SD* = 290 ms; unrelated: *M* = 1466 ms, *SD* = 277 ms; paired sample t-test: *t*(17) = 8.25, *p* < .001.

To obtain further support for our claim that our manipulation specifically hindered memory for CW, we conducted a joint analysis of Experiments 4a and 4b with the equivalent Experiments in which the task directed participants to the semantic statues of the stimuli (Experiments 1 and 3). To enable comparison between different experiments with various FA rates, FA were subtracted from Hit rates (We used Hits-FA rates and not a d' measure because d' rest upon the assumption equal variances for related/congruent and unrelated/incongruent distributions [32], an assumption which was unwarranted in light of the results of Experiment 1 and 3). These were entered to a mixed model ANOVA with Type of Relation (congruency/relatedness) and Task (attention to semantic relations/attention to lexical relation) as between-subject factors and Judgment (CW/R and corrected K) and Status (congruent/related or incongruent/unrelated) as within-subject factors. A triple interaction of Task with Judgment and Status would support the claim that our task differentially influenced CW effect and corrected K (lack of) effect between congruent (and related) and incongruent (and unrelated) items. Indeed, such an interaction was observed (*F*(1,66) = 58.31, *MSE* = .005, *p* < .001). To assure that the triple interaction stemmed from the task-alteration influencing CW effect, an ANOVA with CW/R responses as the dependent measure was conducted (similarly to the principle ANOVA, also here FA rates were subtracted). This ANOVA included as independent variables the between-subject factors of Type of Relation (congruency/relatedness) and Task (attention to semantic relations/attention to lexical relation) and the within subject factors of Status (congruent/related or incongruent/unrelated). We expected to find an interaction of Task with Status in the CW ANOVA (indicating that task modified the CW difference between congruent/related and incongruent/unrelated). Indeed, this interaction was found (*F*(1,66) = 164.91, *MSE* = .003, *p* < .001). There was no interaction of Type of Relation with task and Status, *p* > .48. We conducted the same ANOVA using corrected K rates as dependent measure. No interaction of Task and Status was evident, *p* > .68, indicating that our task did not influence corrected K rates.

We note also that in the former principle ANOVA, (with Type of Relation and Task as between-subject factors and Judgment and Status as within-subject factors), Type of Relation factor interacted with Judgment factor (*F*(1,66) = 6.89, *MSE* = .01, *p* < .05), indicating that items appearing in noun-noun pairs achieved different memory rates than items previously appearing in noun-adjective pairs. Importantly, no four-way interaction of all factors, nor a triple interaction of Task, Type of Relation and Judgment were obtained, indicating that our manipulation similarly affected relatedness and congruency (*p's* > .29).

**Discussion**. In Experiment 4, we employed an identical paradigm to that used in Experiment 1 and 3, with the major difference that we used an encoding task which was meant to specifically hinder item-context integration. As predicted by the integration-elaboration account, the disturbance to the integration of item and context specifically diminished participants' recollection of congruent context words, and the overall mnemonic advantage for congruent stimuli was not obtained. According to the item strength account, hindering context-word retrieval should not have significantly influenced the congruency advantage. Therefore, these findings further support the claim that the congruency effect indeed hinges upon integration, in accordance with the integration account.

Note that these results should be interpreted with a measure of caution due to the low levels of CW responses. Our aim in this experiment was to examine integration as a crucial factor in the congruency effect. Thus, we chose a manipulation that diminished integration. While demonstrating our claim, this manipulation is limited by the relatively low rates of integrated items it produced in both conditions. Further research may provide additional paradigms to investigate this issue.

## General Discussion

Despite the many years that have passed since the first demonstration of the classic congruency effect [3,5], two open questions still surround this effect. First, previous investigations of the effect implicitly adopted the ‘proposition hypothesis’, namely, that the congruency effect strictly pertains to subject-predicate propositions. Still, the validity of this hypothesis has not been investigated. Second, the most prominent explanation of this robust phenomenon—the integration-elaboration hypothesis [3]—lay on equivocal empirical grounds. In the current investigation, we set forth to address these two important issues.

### The role of item-context integration

As noted in the introduction, a central tenant of the integration-elaboration account of the congruency effect hinges upon enhanced item-context integration. The integration of the item with the context words entails that the mental representation of an item becomes more "enriched" or "elaborated", such that it is subsequently better remembered [3].

The assumption that a congruent item-context should somehow be better integrated appeals to intuition. However, its explanatory power to the congruency effect has been as compatible with existing data as that of the item-strength account. The current set of experiments provided strong evidence in support of the item-context integration account. Thus, in Experiments 1 and 3, we applied a modified remember-know paradigm in which participants were asked to report the context word associated with an item. As predicted, target items resulted in enhanced recognition when appearing at encoding within a congruent proposition than when appearing within an incongruent proposition. Importantly, this enhanced recognition was accompanied by better retrieval of the context word. This result provides direct evidence to the claim that congruent word pairs are indeed better integrated, in the sense that the presentation of the target items entails the retrieval of the context word. These findings provide the first demonstrations of enhanced congruency-related item-context integration when the target is retrieved (in contrast to conditions wherein the target word is given as in cued-recall tests). Therefore, they demonstrate the function of integration within the setting in which congruency is typically studied—when target's retrieval is examined.

It is important to note that the integration-elaboration hypothesis is committed to a stronger argument, according to which the congruency effect should specifically hinge upon enhanced item-context integration. This claim gained initial support in Experiment 1 and 3 where it was shown that the congruency advantage for the retrieval of the context words was not accompanied by increased item-only memory (i.e., retrieving the item word without additionally retrieving the context word). Although not ruling out the item strength hypothesis these results suggest that the congruency advantage is specifically manifest in a facilitated ability to retrieve the context word, rather than a generalized memory enhancement of the target (see Fig. 1).

Finally, in order to provide further support for the claim that the congruency effect indeed hinges upon item-context integration, we wanted to see whether an experimental manipulation that hinders this integration eliminates the congruency advantage. Thus, in Experiment 4A and 4B we used an encoding task that specifically hindered context retrieval. As predicted by the integration-elaboration hypothesis, this manipulation reduced context-word retrieval, and significantly reduced the congruency effect (abolishing it in the recognition total memory measure, and leaving a minor difference in the item+context bin). The alternative item-strength hypothesis would have predicted a different pattern of results: since it is not committed to the locus of the effect (in the CW and/or in the item-only bin), in the absence of context integration the congruency advantage could still have appeared in the item-only bin; However, this was not the case.

Fisher and Craik [23] presented evidence that is consistent with the claim that congruency hinges upon item-context integration, rather than item-strength. In their study, the length of a sentence enhanced the retrieval of the target item only if the words in the sentence were associatively related to the target item. The authors maintained that associatively related words can be more easily reinstated at test and concluded that integration at encoding is beneficial only to the extent that it allows the context to be reinstated at retrieval.

Although providing important evidence supportive of the integration account, Fisher and Craik [23] did not gauge memory for the context. Hence, their results are limited in that they cannot distinguish between cases in which the target item served at retrieval to reinstate the context and cases in which it did not, a result which is critical for the integration-elaboration hypothesis. As we have repeatedly argued in the article, it is possible that the enhanced memory of the target item was not related to the reinstatement of the context at retrieval, but rather to the strength of the target item itself, as activated at encoding. One might argue that the second experiment reported in this same study, which exhibited an advantage of the complexity of the sentence only when the full sentence was given at test (that is, reinstated by the experimenter), but not when the target word appeared alone, provides the demonstration that indeed, context reinstatement, rather than the strength of the target item, underlies congruency/relatedness effect. However, as mentioned in Fisher and Craik [23], it is hard to know whether this effect did not simply result from the length of the sentence presented at test. It is thus conceivable that compared to short sentences (or to long sentences containing no associates of the target word), a long sentence in which words were associatively related to the target item activated the target to a greater extent [25] at encoding. For example, the concept DOG which appears in the sentence "The furry barking DOG" will receive an activation boost at encoding from both "barking" and "furry". Thus, it is bound to be better remembered than when appearing within the sentence "The clean black DOG", for which no additional boost is expected from the associatively unrelated concepts of "clean" or "black".

While our findings show that the congruency advantage is contingent upon item-context integration, it is important to note that in our experiments integration was defined (consistently with previous literature) in a relatively narrow sense—as the ability of an item to serve as cue for the retrieval of a context word. Such a definition regards integration as a behavioral outcome, rather than as a well-defined component of a cognitive mechanism. For example, it is possible that the enhanced integration relies upon spreading of activation in a semantic network (see for example, the PIER-II model, [25]). Alternatively, it could be that spreading of activation in the semantic network facilitates memory via enhancement of an episodic trace (see e.g., SAC model, [26]). Clearly, a full understanding of the congruency phenomenon will require future work to formulate and test models that utilize a more precise instantiation of the notion of integration.

Indeed, the current investigation did not aim to provide a conclusive answer to the question of whether the observed "integration" is sub-served by the propagation of activation within a semantic network (e.g, PIER-II, [25]) or the enhancement of an episodic trace (e.g., SAC, [26]). Still, we believe that one aspect of our results may suggest a crucial role for an episodic trace in item-context integration. In both Experiments 1 and 3 (wherein participants reported the context word) there were practically no cases in which participants retrieved an incorrect context word; i.e., there were no cases in which participants falsely recollected that the word "cat" appeared with "dog" when "cat" did not appear at all; nor were there cases where they falsely attributed the word "cat" to the item "dog", when in actuality "cat" appeared with the word "milk".

This finding is particularly striking given the obvious potential for such mistakes. While there are clearly regularities in the participants' dominant semantic associates, there is also much variability. For example, while many people might think of the word "dog" when prompted with the word "cat", others may think of the word "fur". If item-context integration entails the spreading of activation within the semantic network, one could have speculated that at least in some cases, participants will mistake such semantic associates for the context word displayed at encoding. The marked distinctiveness of congruent item-context pairs, suggests that they were encoded in a qualitatively "privileged" manner, perhaps via the enhancement of an episodic trace. This result resonates with previous studies demonstrating prior knowledge influences on episodic memory using source memory or recollection measures [7,36,44–47].

Future work may benefit from applying a computational modeling methodology to ascertain whether such models as PIER-II [25] and SAC [26] indeed diverge in their predictions regarding the existence/proportion of erroneously recalled context words (in a congruency paradigm). Such evidence will be especially important in evaluating a recent influential neuroscientific model of schema congruency and cortical-hippocamapal interaction [48] which suggests that events that are congruent with existing knowledge are encoded via a cortical/semantic route while incongruent events are encoded via a hippocampal/episodic route. Recent neurocognitive studies relevant to the evaluation of this model have thus far yielded inconclusive data: In support of a semantic/cortical item-context integration, some studies found cortical involvement in encoding of congruent but not incongruent items [4,6,7,12]. However, other findings suggest that the hippocampus is required also for existing-knowledge-supported new learning [16,49].

The finding whereby participants did not report any erroneous context-words also pertains to the ongoing debate as to whether source memory judgments (and Remember responses) reflect episodic recollection, or whether they stem from some sort of familiarity judgment [33,34,50]. Much of the previous investigations of source memory entailed presenting participants with a forced-choice question (i.e., items were presented at encoding on the right or left side of the screen, and participants had to arbitrate between these options upon retrieval of the items). Using such paradigms, source memory judgments could have been made by generating the alternatives for the target item (i.e., imagining the word as being displayed on the right or left side of the computer) and then responding based upon a sense of familiarity (i.e., generate-to-recognize, see Taylor & Henson, 2012). In contrast, in the current experiments, a generate-to-recognize strategy should have resulted in at least moderate rates of context word FAs: Because the related word appearing in the experiment was not necessarily the strongest associate of the target word, some semantically-related generated associates should have been retrieved. Therefore, the high accuracy of source memory in our paradigm might strengthen the view that retrieval of source memory reflects recollection of the episodic event (see also [50], for a consistent finding and a similar interpretation, utilizing conceptual priming at retrieval).

### The role of propositional structure

The second major concern dealt with in the current investigation pertained to the question of what determines whether an item and context will be integrated or not. Past demonstrations of the congruency effect have made use of item-context pairs (in themselves, or embedded within longer sentences) that comprise subject-predicate propositions that have the property of being veridical or not. False or insensible propositions (e.g., “ecstatic spinach”) differ from true/sensible ones (“leafy spinach”) in two aspects. First, they violate our knowledge of the world. Second, they are composed of semantically unrelated elements. According to our account, it is the latter factor of semantic relatedness that constitutes the congruency phenomenon. This claim received strong support throughout the experiments described in this article.

Specifically, in Experiment 2 we showed that a difference in memory performance of a similar magnitude could be also observed with word pairs that do not form subject-predicate propositions (e.g., “BEACH-bikini”) but rather merely have the property of being semantically related (or unrelated). Notably, this relatedness effect was observed in a paradigm similar to previous investigations of the congruency effect, and different from investigations of associative memory (see Introduction to Experiment 2). Thus, Experiment 2 proved the feasibility of our semantic relatedness account. The suggestion that the advantage for true/sensible and for semantically related items stem from the same underlying mechanism received further support when examining the combined pattern of results from Experiments 1, 3 and 4. Experiments 1 and 4A showed that the use of a more sensitive measure of memory performance ("remember-know" judgments) uncovers a more intricate and specific pattern of results that sub-serves the congruency effect: this effect seems to stem from a significant advantage in the ability to recall the encoding context, but not in an advantage of item-only retrieval (i.e., retrieval of the item without additionally retrieving the context word). Importantly, this relatively specific pattern of results appeared both when for true/sensible and false/insensible propositions (Experiment 1) and for related/unrelated word pairs (Experiment 3). Furthermore, a manipulation that reduced the overall memory performance (Experiment 4A and 4B) once again produced a similar pattern of results for true/false propositions and for related/unrelated word pairs. Finally, a joint analysis of Experiments 1, 3, 4A and 4B revealed that the factor of Type of Relation (i.e., congruency or relatedness) did not interact with neither Task (attention to semantic relations/attention to lexical relation), Status (congruent/related or incongruent/unrelated) or with the combination of the two factors. This analysis provides further support to the claim the same mechanism gives rise to the congruency and the relatedness mnemonic advantage.

Other than delineating a classic and robust memory phenomenon, we believe that the current investigation speaks to a wider discussion in the field of learning and memory, regarding the role of associative vs. propositional processes. Craik and Tuvling's [3] classic paper on semantic congruency, depth of processing, and elaboration, (implicitly) adopted the principles of formal-propositional logic to explain what constitutes a congruent item-context pair. Furthermore, it attempted to explain the congruency phenomenon in terms of domain-specific processes (i.e., elaboration, distinctiveness). More recent models of memory (e.g., [26,30,51]) have strived to provide a unified account of diverse memory phenomena by assuming that they are the result of a basic structure of interconnected nodes that operate according to a few underlying domain-general principles (e.g., the spreading of activation principle, e.g., [52,53], or the idea that neurons/nodes that "fire together", "wire together", e.g., [54]). These models have become triumphant in current literature, due to their relative parsimony and impressive explanatory power.

And yet, the success of associationist theories of learning and memory does not mean that propositional accounts of cognition have become irrelevant. For example, in the field of human associative learning, there is an ongoing debate regarding whether our knowledge of contingencies is propositional or association-based (see [55], for a review). Thus, it appears that the dialectic relationship between the associationist and propositional views of knowledge may be here to stay. Hopefully, continued research along this path might assist us in producing a more refined and accurate understanding of memory phenomena.

### What is the role of prior knowledge in memory for new experiences?

The congruency effect is a notable member of a family of memory phenomena that show that our prior knowledge has a crucial role in the formation of new memories. In that sense, the congruency effect might be a variant of classic studies that have shown we tend to remember better information that is consistent with our existing knowledge schemas (see, for example, [1,2]).

A central task of an organism is to master the structure of objects in its environment and the contingencies that govern aversive and appetitive consequences. In order to attain this goal, cognition must be able to identify new events as instances of existing schemas [56,57]. Therefore, one might expect that whenever this basic impetus of cognition goes unanswered, memory will falter. From this perspective, it is clear why familiar stimuli (i.e., “yellow banana”) are better remembered than stimuli which are incongruent with our prior knowledge, (i.e., a “purple banana”).

And yet, to efficiently and flexibly adapt to changing environments, the detection and processing of novel events is no less important. Indeed, much work shows that humans are novelty-seekers, or “Infovores” [58]. Novel items have been shown to draw our attention [59–62]. Critically, in some cases, novel items are better remembered than familiar ones (e.g. [63–66]. In the case of congruency, coming across a purple banana is a novel occurrence that may be more informative than seeing yet another yellow banana. In light of this consideration, it could have been expected that a purple banana will be better remembered than yet another yellow banana.

Theories of human memory will have to improve our understanding of the complex interplay between the opposing forces of familiarity and novelty (for recent attempts to reconcile this appearing tension, see [15,46,48,67]). In other words, we will have to understand the principles that govern whether knowledge that is incongruent with an existing model of the world is rejected or accommodated. To that end, we believe that continued research into the congruency effect may be invaluable.
